# Supplementary material for: Research on Selected Wildlife Infections in the Circumpolar Arctic—A Bibliometric Review
Source: Int J Environ Res Public Health. 2022 Sep 7;19(18):11260. doi: 10.3390/ijerph191811260 (PMC9517571; doi:10.3390/ijerph191811260)
Supplement: Supplementary file 1 [file ijerph-19-11260-s001.zip › File S2_23082022_clean_AE.pdf]

File S2

Review of publications concerning fish parasites and infections

There were two different main types of publications concerning fish parasites and infections in our scoping review, one focusing mainly on aquatic ecology or research between fish populations, and another diverse group about prevalence and disease agents in fishes, which is shortly introduced below.

In many cases, the reduction in population density of the final host controls the pathogen. The distribution and co-occurrence and exchange of adult cestode species in fish communities were studied in the Bothnian Bay and Lake Yli-Kitka in Finland, and coexistence of two or more mature cestode species was not found in the majority of fish species studied (31 of 33; 94%) [1]. The circulation of 27 parasite taxa among 12 species of fish from lakes showed that most of the parasite taxa were host specialist in the Caribou Mountains in Alberta [2]. Many have birds as definitive hosts, while *Triaenophorus crassus* infection circulates between whitefish *Coregonus lavaretus* and pike *Esox lucius* [3]. The prevalence and infection intensity of the bird tapeworm *Diphyllobothrium ditremum* plerocercoids in trout *Salmo trutta* were found after establishment of European minnow *Phoxinus phoxinus* in a subalpine lake in Norway [4] . Two lakes with and without sympatric burbot *Lota lota* and another six fish species in subarctic Norway were studied by Knudsen et al. [5]. They found that dense populations of burbot may have very strong impacts on the ecology and evolution of arctic charr *Salvelinus alpinus* populations, possibly stronger than impacts from other sympatric species. The role of the feeding behavior of fish hosts in Northern Norway studied in arctic charr strongly showed the commonly observed aggregation of helminths among hosts under certain conditions. The association of parasite species *Cyathocephalus truncatus, C. farionis, Diphyllobothrium dendriticum,* and *D. ditrenum* and fish stomach contents were studied [5]. Kuhn et al. have published about *Diphyllobothrium* spp. infections in brown trout and sticklebacks *Pungitius pungitius* play a key role in the transmission of *Diphyllobothrium* spp. parasite larvae [6], and new methodology was developed [7].
Charrs with parasites had cannibalism, and twelve species of fresh-water parasites were found in a river-lake system in Uzon Caldera Kamchatka [8]. Cannibalism was found to result in accumulation of cestodean parasites and is an important strategy for survival of arctic charr. However, it reduced the survival rate of old individuals in autonomous lake ecosystems in Svalbard [9]. Fish prey may be the most important source for *Diphyllobothrium* spp. infections in trout, whereas charr predominantly acquire *Diphyllobothrium* spp. by feeding on copepods in Northern Norway [10]. Researchers also found interspecific and intraspecific (extent of cannibalism) host behavioral interactions. In an isolated lake in Kamchatka, there were 29 species of parasites found in charrs, indicating differences in preference for food items and occupied biotopes and supporting the ecological differentiation of charrs [11].

Garnick and Margolis [12] examined the presence of helminth parasites in sockeye salmon (*Oncorhynchus nerka*) smolts in Vancouver Island and found the parasite infection may account to some extent for variability observed in the migratory behavior of smolts. The latitudinal variations may be explained in terms of life cycles and endemic areas of the four studied parasite species in cod along the west coast of Norway, and they may be used as biological tags of cod and other gadoid fish [13].

An increased amount of *Diphyllobothrium nihonkaiense* infected wild pink salmon (*Oncorhynchus gorbuscha*) was found near Hope in Alaska [14]. It was found that *D. dendriticum* larvae as a cause of heart disease (massive chronic myocarditis) lead to mortality in hatchery-reared sea trout and brown trout in a freshwater fish farm in Northern Finland, and the occurrence of 4 cestode larval species (100% infected, possible sources: vendace *Coregonus albula*, whitefish and nine-spined stickleback) decrease the commercial value of brown trout in a large regulated lake in Northern Finland [15]. Among Russian papers, one provided data on Northern Finland. It studied parasite diversity in Salmonidae spp. fish from the Utsjoki River, obtained during 1993-1995 and 2006-2007. Twenty species of salmon parasites were found, the majority of which are parasites with complicated life cycles. Brown trout had the highest number of specific parasite species [16].

Most of the Russian publications (n=13) from e.Library were from Karelia, European North of Russia, and parasites from wild fish were reported studied in five lakes and three rivers [17–29]. The researchers found 42 species of parasites among fish species in the lakes Onega and Ladoga. The results indicate that the fish are related not only taxonomically, but also ecologically [24]. The only exception was the acanthocephalan *Corynosoma semerme* found in trout from Ladoga. Being a sea relict, the parasite serves as evidence of former relations between the Ladoga lake and the Baltic Sea [24,19]. High similarity was also found specifically between the cestode faunas in the fish of the Baltic and the Arctic oceanic provinces [28].

Ieshko et al. presented data about the population dynamics of glochidia of the freshwater pearl mussel *Margaritifera margaritifera*, a declining species of European fauna, parasitizing on juvenile Salmonidae spp. fishes in rivers of northern Europe. In addition, they recorded for the first time the alien species *Gyrodactylus salaris* in salmon of the Pista river In the Keret' River (White Sea basin), *G. salaris* was found almost every year since 1991 in mostly juvenile Atlantic salmon, decreasing the population size [21]. The other threats for pearl mussel and salmon populations were poaching, incorrect strategy of artificial reproduction of salmon, the *Gyrodactylus* parasite, introduced pink salmon, and uncontrolled tourism [27].

Dorovskikh [30–35] and Dorovskikh and Stepanov [36] studied fish in the river of Northern Dvina and its tributary Mesen, Kama and Vychegda in the Arkhangelsk region, as well as the river Pechora in the republic of Komi. He/they summed up the results of the 70 years' long study of a species composition in the basins of the rivers in local fish:

- 56 species of trematode parasites were recorded in the examined region [30]
- 66 ichthyoparasite protozoan species [31]
- 86 monogenean species [32]
- 21 nematode species and 9 acanthocephalan worm species [33]
- 26 cestode species [34]
- 4 leech species, 5 mollusk species, 16 crustacean species and 1 water mite species [35]
- and, most recently, parasite fauna of the minnow was described [36].

In Murmansk Oblast, Kola Bay of the Barents Sea, Kuklin et al. studied species composition and seasonal dynamics of the bullroat *Myoxocephalus scorpius* helminthofauna [37]. They presented 18 species (8 nematodes, 5 trematodes, 3 cestodes, and 2 acanthocephalans). The maximum species variety was

recorded during the winter period. The seasonal appearance of bullroat parasites that use sea mammals as final hosts was found and discussed [37]. Tkachenko et al. researched the infestation status of Atlantic salmon *Salmo salar* with *Anisakis simplex* larvae in the river Ponoi; they found the level and dynamics of infestation of autumn Atlantic salmon in 2009-2016 and compared this information with the historical data. The results have shown that the proportion of salmon infested with parasite varied from 2-60%, and the number of parasites in a single fish varied from 1 to 28 specimen. The studied Red Vent Syndrome (RVS) has never been confirmed for Ponoi Atlantic salmon. The assumption has been made that the level of infestation depends mostly on the role of different salmon food species in the parasite life cycle and their availability in different years as well as salmon migration routes in the ocean [38].

In the Yamal and Gydan peninsula, there were two articles about parasites of whitefish, arctic loach *Orthrias* sp., and salmon. Seventeen taxa of parasites were found in whitefish and arctic loach that were similar to other circumpolar fish, depending on fish migratory routes and diet [39,40]. Further east in large water reservoirs of Yakutia, three articles focused on parasites in fish. Seventeen species of fish had cestodes *Triaenophorus* spp. at a prevalence of: *T. nodulosus* 22–42% with an intensity of infection of 13–23 parasites per fish, and *T. crassus* – 5–93% with an intensity of infection of 7–14 parasites per fish [41]. Fourteen species of fish and 45 species of parasites were recorded for the Ungra River. Myxosporeans, infusoria, monogeneans, and trematodes were found dominant in minnows, while at the same time nematodes were predominant in the arctic grayling *Thymallus* sp. [42]. The most common parasitic disease of the crucian carp *Carassius carassius* was myxosporidiosis caused by the genus *Myxobolus*, *Digramma* sp.(a Cestode), and *Philometra* sp. nematode infections [43].

References

1. Andersen, K.I.; Valtonen, E.T. On the infracommunity structure of adult cestodes in freshwater fishes. *Parasitology* **1990**, *101 Pt 2*, 257–264, doi:10.1017/s0031182000063319.
2. Baldwin, R.E.; Goater, C.P. Circulation of parasites among fishes from lakes in the Caribou Mountains, Alberta, Canada. *J. Parasitol.* **2003**, *89*, 215–225, doi:10.1645/0022-3395(2003)089[0215:COPAFF]2.0.CO;2.
3. Amundsen, P.-A.; Kristoffersen, R. Infection of whitefish (*Coregonus lavaretus* L. s.l.) by Triaenophorus crassus Forel (Cestoda: Pseudophyllidea): A case study in parasite control. *Can. J. Zool.* **1990**, *68*, 1187–1192, doi:10.1139/z90-176.
4. Borgstrøm, R.; Trømborg, J.; Haugen, T.O.; Rosseland, B.O. Plerocercoids of the cestode *Diphyllobothrium ditremum* in brown trout *Salmo trutta*: Substantial increase in infection after establishment of European minnow *Phoxinus phoxinus*. *J. Fish Biol.* **2017**, *91*, 912–927, doi:10.1111/jfb.13391.
5. Knudsen, R.; Amundsen, P.-A.; Klemetsen, A. Arctic charr in sympatry with burbot: Ecological and evolutionary consequences. *Hydrobiologia* **2010**, *650*, 43–54, doi:10.1007/s10750-009-0077-2.
6. Kuhn, J.A.; Frainer, A.; Knudsen, R.; Kristoffersen, R.; Amundsen, P.-A. Effects of fish species composition on *Diphyllobothrium* spp. infections in brown trout—Is three-spined stickleback a key species? *J. Fish Dis.* **2016**, *39*, 1313–1323, doi:10.1111/jfd.12467.
7. Kuhn, J.A.; Knudsen, R.; Kristoffersen, R.; Amundsen, P.-A. A simplified method to estimate *Diphyllobothrium* spp. infection in salmonids. *J. Fish Dis.* **2017**, *40*, 863–871, doi:10.1111/jfd.12566.
8. Busarova, O.Y.; Esin, E.V. Parasite fauna of landlocked Dolly Varden (Salvelinus, Salmonidae) from the river–lake system of Uzon caldera (Kamchatka). *J. Ichthyol.* **2015**, *55*, 933–936. Available online: https://agris.fao.org/agris-search/search.do?recordID=US201600089478 (accessed on 12 March 2021).
9. Hammar, J. Cannibals and parasites: Conflicting regulators of bimodality in high latitude Arctic char, *Salvelinus alpinus*. *Oikos*, **2000**, *88*, 33–47. doi:10.1034/J.1600-0706.2000.880105.X.
10. Henriksen, E.H.; Knudsen, R.; Kristoffersen, R.; Kuris, A.M.; Lafferty, K.D.; Siwertsson, A.; Amundsen, P.-A. Ontogenetic dynamics of infection with *Diphyllobothrium* spp. cestodes in sympatric Arctic charr *Salvelinus alpinus* (L.) and brown trout *Salmo trutta* L. *Hydrobiologia* **2016**, *783*, 37–46, doi:10.1007/s10750-015-2589-2.
11. Busarova, O.Y.; Knudsen, R.; Markevich, G.N. Паразитофауна гольцов (salvelinus) озера Кроноцкое, Камчатка / Parasites fauna of the lake Kronotskoe charrs (salvelinus), Kamchatka. *Паразитология* **2016**, *50*, 409–425.

12. Garnick, E.; Margolis, L. Influence of Four Species of Helminth Parasites on Orientation of Seaward Migrating Sockeye Salmon (Oncorhynchus nerka) Smolts. *Can. J. Fish. Aquat. Sci.* **1990**, *47*, 2380–2389, doi:10.1139/f90-265.
13. Hemmingsen, W.; Mackenzie, K. Latitudinal variations in the occurrence of some cod parasites along the west coast of Norway. *Mar. Biol. Res.* **2013**, *9*, 431–436. doi:10.1080/17451000.2012.742548.
14. Kuchta, R.; Oros, M.; Ferguson, J.; Scholz, T. *Diphyllobothrium nihonkaiense* Tapeworm Larvae in Salmon from North America. *Emerg. Infect. Dis.* **2017**, *23*, 351–353. doi:10.3201/eid2302.161026.
15. Rahkonen, R.; Koski, P. Occurrence of cestode larvae in brown trout after stocking in a large regulated lake in northern Finland. *Dis. Aquat. Org.* **1997**, *31*, 55–63, doi:10.3354/dao031055.
16. Ieshko, E.P.; Barskaya, Y.Y.; Lebedeva, D.I.; Novokhatskaya, O.V.; Kaukoranta, M.; Niemela, E. Особенности паразитофауны молоди атлантического лосося (salmo salar l.), форели (salmo truttae l.) и гольца (salvelinus alpinus l.) системы реки Утсйоки (северная Финляндия)/Peculiarities of the parasitofauna of Atlantic salmon parr (*Salmo salar* L.), brown trout (*Salmo truttae* L.), and char (*Salvelinus alpinus* L.) in the Utsjoki river system (northern Finland). *Паразитология* **2011**, *45*, 26–36.
17. Sidorova, N.A.; Parshukov, A.N. Условно-патогенная микрофлора в рыбоводных хозяйствах Карелии / Diversity of conditionally pathogenic microorganisms in fish industry facilities in Karelia. *Хранение И Переработка Сельхозсырья* **2010**, *6*, 14–16.
18. Anikeeva, L.V.; Ieshko, E.P.; Rumyantsev, E.A. Экологический анализ гельминтов ряпушки и корюшки онежского озера / Ecological analysis of helminths in vendace and smelt from the Onega lake. *Труды Карельского Научного Центра Российской Академии Наук* **2016**, *4*, 37–47.
19. Rumyantsev, E.A. Фауна паразитов лососевых рыб (салмониде, пискес) Онежского и Ладожского озер / Fauna of parasites of salmon fishes (salmonidae, pisces) of the Onega and Ladoga lakes. *Наука И Мир* **2014**, *1*, 95–98.
20. Evseeva, N.V.; Ieshko, E.P.; Shulman, B.S. Роль акклиматизации в формировании паразитофауны европейской корюшки в условиях Сямозера (Карелия) / The role of acclimatisation in the formation of parasite fauna in the European smelt osmerus eperlanus in the Sjamozero lake (Karelia). *Паразитология* **1999**, *33*, 404–409.
21. Artamonova, V.S.; Makhrov, A.A.; Shulman, B.S.; Khaimina, O.V.; Lajus, D.L.; Yurtseva, A.O.; Shirokov, V.A.; Shurov, I.L. Реакция популяции атлантического лосося Salmo Salar l. реки Корить на инвазию паразита gyrodactylus salaris malmberg / Response of the Atlantic salmon salmo salar population of the Keret' river on the invasion of parasite gyrodactylus salaris malmberg. *Российский Журнал Биологических Инвазий* **2011**, *4*, 2–14.
22. Ieshko, E.P.; Larson, B.M.; Pavlov, Y.L.; Barskaya, D.I.; Lebedeva, D.I.; Novokhatskaya, O.V. Популяционная динамика численности глохидий пресноводной жемчужницы margaritifera margaritifera l., паразитирующих на молоди лососевых рыб северных водоемов / Population dynamics of the number of glokhidia of freshwater pearls *Margaritifera margaritifera* L., parasitiating on young salmon fishes of northern reservoirs. *Известия Российской Академии Наук Серия Биологическая* **2009**, *6*, 734–739.
23. Tyrkin, I.A.; Shustov, Y.A.; Rasputina, E.N.; Legun, A.G. Особенности питания молоди атлантического лосося (salmo salar l.), заражённой инвазионным паразитом gyrodactylus salaris, в реке Кереть / Feeding peculiarities in Atlantic salmon fry (salmo salar l.) infected with invasive parasite gyrodactylus salaris in river Keret. *Российский Журнал Биологических Инвазий* **2015**, *8*, 71–76.
24. Mamontova, O.V. Особенности паразитофауны форели (Salmo trutta morpha lacustris L.) в озерах Карелии / Characteristics of the parasite fauna of brown trout (Salmo trutta morpha lacustris L.) in lakes of Karelia. *Ученые Записки Петрозаводского Государственного Университета* **2016**, *4*, 72–75.
25. Mamontova, O.V. Особенности паразитофауны ряпушки европейской Coregonus albula Ладожского озера / Parasitofauna features of the Ladoga lake vendace coregonus albula. *Ученые Записки Петрозаводского Государственного Университета* **2017**, *6*, 78–81.
26. Shulman, B.S.; Shurov, I.L.; Shirokov, V.A. Некоторые особенности биологии и паразитофауны арктического гольца (salvelinus alpinus linnaeus) оз. Мушталампи (северная Карелия) / Some features of the biology and parasite fauna of the arctic char (salvelinus alpinus l.) in the lake Mushtalampi (northern Karelia). *Паразитология* **2016**, *50*, 325–330.
27. Zyuganov, V.V. Мониторинг биосистемы "жемчужница лосось" в карельской реке Кереть за последние 17 лет / Monitoring of the biosystem "Pearl salmon" in the Karelian River Keret' for the last 17 years. *Успехи Современной Биологии* **2008**, *128*, 424–430.
28. Anikieva, L.V.; Rumyantsev, E.A. Вопросы формирования и зоогеографии фауны цестод рыб озер Карелии / On the formation and zoogeography of cestode fauna in fishes inhabiting lakes of Karelia. *Труды Карельского Научного Центра Российской Академии Наук* **2008**, *13*, 17–25.
29. Ieshko, E.P.; Schurov, I.L.; Shulman, B.S.; Barskaya, Y.Y.; Lebedeva, D.I.; Shirokov, V.A. Особенности биологии, паразитофауны и встречаемости опасного паразита gyrodactylus salaris malmberg, 1957 на молоди пресноводного лосося (salmo salar m. sebago girard) реки Писта (бассейн Белого моря) / Peculiarities of the biology and parasite fauna of juvenile Atlantic salmon (salmo salar l.) in the Pista river (White Sea basin), according to the gyrodactylus salaris infestation. *Паразитология* **2012**, *46*, 279–289.
30. Dorovskikh, G.N. Итоги изучения видового состава паразитов рыб бассейнов рек северо-востока Европейской России. Трематоды (Trematoda) / Results of the study of species composition of fish parasites in river basins of the north-east European Russia. Trematoda. *Паразитология* **1997**, *31*, 551–564.
31. Dorovskikh, G.N. Итоги изучения видового состава паразитов рыб бассейнов рек северо-востока Европейской России. Простейшие / Results of the study of species composition of fish parasites in river basins of the north-east European Russia. Protozoans. *Паразитология* **1997**, *31*, 296–306.

32. Dorovskikh, G.N. Итоги изучения видового состава паразитов рыб бассейнов рек северо-востока Европейской России. Моногенеи (Monogenea) / Results of the study of species composition of fish parasites in river basins of the north-east European Russia. Monogenea. *Паразитология* **1997**, *31*, 427–437.
33. Dorovskikh, G.N. Итоги изучения видового состава паразитов рыб бассейнов рек Северо-Востока Европейской России. Нематоды (Nematoda) и скребни (Acanthocephala) / Results of the study of species composition of fish parasites in river basins of the north-east European Russia. Nematoda, Acanthocephala. *Паразитология* **1999**, *33*, 446–452.
34. Dorovskikh, G.N. Итоги изучения видового состава паразитов рыб бассейнов рек северо-востока Европейской России. Цестоды (Cestoda) / Results of the study of species composition of fish parasites in river basins of the north-east European Russia. Cestoda. *Паразитология* **2000**, *34*, 441–446.
35. Dorovskikh, G.N. Итоги изучения видового состава паразитов рыб бассейнов рек северо-востока Европейской России. Пиявки (Hirudinea), Моллюски (Mollusca), Раки (Crustacea), Паукообразные (Arachnida) / Results of the study of species composition of fish parasites in river basins of the north-east European Russia. Hirudinea, Mollusca, Crustacea, Arachnida. *Паразитология* **2000**, *34*, 158–163.
36. Dorovskikh, G.N.; Stepanov, V.G. Итоги изучения географической изменчивости паразитофауны и структуры компонентных сообществ паразитов гольяна phoxinus phoxinus (l.). 1. Бассейны рек Камы и С.Двины / Results of the study of the geographical variability of the parasite fauna and of the structure of component communities of the minnow parasite phoxinus phoxinus (l.) 1. Basins of the Kama and Northern Dvina rivers. *Паразитология* **2013**, *47*, 113–122.
37. Kuklin, V.V.; Kuklina, M.M.; Kisova, N.E. Видовой состав и сезонная динамика гельминтофауны европейского керчака (myoxocephalus scorpius, cottidae) в Кольском заливе Баренцева моря / Species composition and seasonal dynamics of the helminthofauna of the bullroat (myoxocephalus scorpius, cottidae) from Kola Bay of the Barents sea. *Зоологический Журнал* **2012**, *91*, 131–137.
38. Tkachenko, A.V.; Prusov, S.V.; Karasev, A.V.; Shkatelov, A.P. Современное состояние зараженности атлантического лосося р. Поной (Мурманская область) личинками нематоды Anisakis simplex / Current infestation status of Atlantic salmon with Anisakis simplex larvae in the river Ponoi (the Murmansk region). *Вестник Мурманского Государственного Технического Университета* **2017**, *20*, 455–462.
39. Gavrilov, A.L. Паразиты сиговых рыб и Арктического гольца полуострова Ямал / Parasites of whitefish and Arctic loach of the Yamal peninsula. *Научный Вестник Ямало-Ненецкого Автономного Округа* **2000**, *4-2*, 23–26.
40. Selyukov, A.G.; Shuman, L.A.; Nekrasov, I.S. Состояние гонад у лососевидных рыб в субарктических озерах Ямала и Гыдана / Condition of gonads in salmon fish in the subarctic lakes of Yamal and Gydana. *Вестник Тюменского Государственного Университета Экология И Природопользование* **2012**, *6*, 31–40.
41. Odnokurtsev, V.A.; Apsolihova, O.D.; Reshetnikov, A.D. Зараженность рыб цестодами семейства Triaenophoridae Loennberg, 1889 в водоемах Якутии / Contamination of fishes by cestodes family Triaenophoridae Loennberg, 1889 in water reservoirs of Yakutia. *Российский Паразитологический Журнал* **2009**, *1*, 5–9.
42. Butorina, T.E.; Reznik, I.V.; Korneev, A.A.; Glushak, D.V. Ихтиофауна и паразиты рыб реки Унгра (Саха Якутия) / Ichthyofauna and fish parasites of the Ungra river (Sakha Yakutia). *Научные Труды Дальневосточного Государственного Технического Рыбохозяйственного Университета* **2012**, *27*, 3–13.
43. Stepanov, K.M.; Platonov, T.A.; Nyukkanov, A.N.; Kuzmina, N.V. Пищевая ценность и основные инвазионные заболевания карася Якутского (carassius jacuticus, Kirillov) / Nutrition value and main invasion diseases of Yakut crucian (carassius jacuticus, Kirillov). *Международный Научно-Исследовательский Журнал* **2018**, *6-1 (72)*, 93–96.
