# Supplementary material for: Research on Selected Wildlife Infections in the Circumpolar Arctic—A Bibliometric Review
Source: Int J Environ Res Public Health. 2022 Sep 7;19(18):11260. doi: 10.3390/ijerph191811260 (PMC9517571; doi:10.3390/ijerph191811260)
Supplement: Supplementary file 1 [file ijerph-19-11260-s001.zip › File S3.pdf]

Search Terms Used:

ENGLISH WILDLIFE INFECTION SEARCHES		
**FISH**		
PubMed	(((((arctic regions OR subarctic OR polar OR circumpolar OR alaska OR canada OR canadian arctic OR greenland OR iceland OR norway OR svalbard OR faroe islands OR sweden OR finland OR lapland OR scandinavia OR fennoscandia OR russia OR russian arctic OR siberia)) AND animal diseases) AND ("1990"[Date - Publication] : "3000"[Date - Publication])) AND (salmon OR salmonidae OR trout OR salmonids OR cod OR gadidae OR esocidae OR pikes OR cyprinidae OR minnow)) AND (anisakidae OR diphyllobothrium OR opisthorchis)	35
Scopus	(salmon OR trout OR salmonidae) AND (anisakidae OR diphyllobothrium OR opisthorchis)	134
	(cod OR gadidae) AND (anisakidae OR diphyllobothrium OR opisthorchis)	36
	(pike OR esocidae) AND (anisakidae OR diphyllobothrium OR opisthorchis)	10
	(cyprinidae OR minnow) AND (anisakidae OR diphyllobothrium OR opisthorchis)	22
		PubMed: 35<br>Scopus: 202<br>PubMed+Scopus = 237<br>Duplicates removed: 31<br><br>FISH TOTAL: 206<br>After 1st review: 39
**BIRDS**		
PubMed	(((((((((arctic regions OR subarctic OR polar OR circumpolar OR alaska OR canada OR canadian arctic OR greenland OR iceland OR norway OR svalbard OR faroe islands OR sweden OR finland OR lapland OR scandinavia OR fennoscandia OR russia OR russian arctic OR siberia)) AND animal diseases) AND ("1990"[PDAT] : "3000"[PDAT])) AND (duck OR gull* OR goose OR grouse OR geese OR anser OR chen OR branta OR somateria OR polysticta OR clangula OR melanitta OR larus OR phasianidae) AND (avian influenza)) NOT chen[Author])	227
Scopus	("avian influenza") AND (gull OR goose OR geese OR grouse OR duck OR anser OR chen OR branta OR somateria OR polystica OR clangula OR melanitta OR larus OR phasianidae) AND (canada OR alaska OR greenland OR iceland OR Norway OR Sweden OR finland OR Russia OR arctic) AND (pubyear > 1989)	165
		PubMed: 227<br>Scopus: 165<br>PubMed+Scopus = 392

Search Terms Used:

		Duplicates removed: 115<br><br>BIRD TOTAL: 277<br>After 1st review: 105
**MAMMALS**		
PubMed	(arctic regions OR subarctic OR polar OR circumpolar OR alaska OR canada OR canadian arctic OR greenland OR iceland OR norway OR svalbard OR faroe islands OR sweden OR finland OR lapland OR scandinavia OR fennoscandia OR russia OR russian arctic OR siberia)) AND animal diseases) AND ("1990"[PDAT] : "3000"[PDAT])) AND (reindeer OR caribou OR rangifer tarandus)	109
	(arctic regions OR subarctic OR polar OR circumpolar OR alaska OR canada OR canadian arctic OR greenland OR iceland OR norway OR svalbard OR faroe islands OR sweden OR finland OR lapland OR scandinavia OR fennoscandia OR russia OR russian arctic OR siberia)) AND animal diseases) AND ("1990"[PDAT] : "3000"[PDAT])) AND (moose OR alces alces)	83
	(arctic regions OR subarctic OR polar OR circumpolar OR alaska OR canada OR canadian arctic OR greenland OR iceland OR norway OR svalbard OR faroe islands OR sweden OR finland OR lapland OR scandinavia OR fennoscandia OR russia OR russian arctic OR siberia)) AND animal diseases) AND ("1990"[PDAT] : "3000"[PDAT])) AND (sled dog OR sledge dog)	21
	(arctic regions OR subarctic OR polar OR circumpolar OR alaska OR canada OR canadian arctic OR greenland OR iceland OR norway OR svalbard OR faroe islands OR sweden OR finland OR lapland OR scandinavia OR fennoscandia OR russia OR russian arctic OR siberia)) AND animal diseases) AND ("1990"[PDAT] : "3000"[PDAT])) AND (brown bear OR ursus arctos OR black bear OR ursus americanus OR polar bear OR ursus maritimitus)	53
	(arctic regions OR subarctic OR polar OR circumpolar OR alaska OR canada OR canadian arctic OR greenland OR iceland OR norway OR svalbard OR faroe islands OR sweden OR finland OR lapland OR scandinavia OR fennoscandia OR russia OR russian arctic OR siberia)) AND animal diseases) AND ("1990"[PDAT] : "3000"[PDAT])) AND (arctic fox or vulpes lagopus)	44
	(arctic regions OR subarctic OR polar OR circumpolar OR alaska OR canada OR canadian arctic OR greenland OR iceland OR norway OR svalbard OR faroe islands OR sweden OR finland OR lapland OR scandinavia OR fennoscandia OR russia OR russian arctic OR siberia)) AND animal diseases) AND ("1990"[PDAT] : "3000"[PDAT])) AND (wolf or canis lupus)	40

Search Terms Used:

	(arctic regions OR subarctic OR polar OR circumpolar OR alaska OR canada OR canadian arctic OR greenland OR iceland OR norway OR svalbard OR faroe islands OR sweden OR finland OR lapland OR scandinavia OR fennoscandia OR russia OR russian arctic OR siberia)) AND animal diseases) AND ("1990"[PDAT] : "3000"[PDAT])) AND (hare OR lepus)	71
	(arctic regions OR subarctic OR polar OR circumpolar OR alaska OR canada OR canadian arctic OR greenland OR iceland OR norway OR svalbard OR faroe islands OR sweden OR finland OR lapland OR scandinavia OR fennoscandia OR russia OR russian arctic OR siberia)) AND animal diseases) AND ("1990"[PDAT] : "3000"[PDAT])) AND (red fox OR vulpes vulpes)	49
		PubMed Total: 458<br>Duplicates removed: 93<br>Total after duplicates removed: 365
Scopus	(TITLE-ABS-KEY ((reindeer OR caribou OR "rangifer tarandus") OR (moose OR "alces alces") OR ("sled dog" OR "sledge dog") OR ("polar bear" OR "brown bear" OR "black bear" OR "ursus arctos" OR "ursus maritimus" OR "ursus americanus")) OR TITLE-ABS-KEY (("arctic fox" OR "vulpes lagopus" OR "red fox" OR "vulpes vulpes") OR (wolf OR "canis lupus") OR (hare OR lepus)) AND TITLE-ABS-KEY ((arctic OR canada OR alaska OR greenland OR iceland OR "faroe islands" OR svalbard OR norway OR sweden OR finland OR lapland OR russia OR scandinavia OR fennoscandia OR siberia)) AND TITLE-ABS-KEY (infection)) AND PUBYEAR > 1989	452
		PubMed: 458<br>Scopus: 452<br>Scopus + PubMed= 910<br>Duplicates removed: 125<br>Total after duplicates removed: 785<br><br>Total: 785<br>After 1st review: 376
		**FISH + BIRDS + MAMMALS= 1551**<br>**After 1st review: 520**

SEARCH WORDS IN ELIBRARY.RU (IN RUSSIAN)

Total amount of library items to search from: 28 498 899 (by 21.01.2019)
Limited to publication types: academic articles, books, dissertations
Years: -
Additional parameters: search in publications with full-text available for the user; search taking morphology into account (analogous word forms)

Search Terms Used:

BIRDS:

Search term in Russian	Matches	Search term translated to English
Птичий грипп	122	Avian influenza
Грипп птиц	295	Avian influenza
**BIRDS TOTAL matches**	**417**	
Selected for initial full-text analysis	3<br>28	in Bird Parasites, 2001 – 2017<br>in Bird Infection/virus, 2005 – 2018
Included in the Article Table (excel) after brief abstract-text analysis	17<br>8	in arctic regions<br>in Russia total/random regions (might be still relevant)<br>2001 - 2018

FISH:

Search term in Russian	Matches	Search term translated to English
Вирусные болезни рыб	15	Viral diseases fish
Инфекционные болезни рыб	32	Infectious diseases fish
Бактериальные болезни рыб	23	Bacterial diseases fish
Паразиты рыб	419	Fish parasites
Инфекции рыб	62	Fish infections
Гельминтозы рыб	9	Fish helminths
Инвазии рыб	162	Fish invasions
Лосось паразиты	25	Salmon parasites
Лосось инфекции	3	Salmon infections
Форель паразиты	2	Trout parasites
Форель инфекции	1	Trout infections
Щука паразиты	28	Pike parasites
Щука инфекции	0	Pike infections
Треска паразиты	6	Cod parasites
Треска инфекции	1	Cond infections
Горбуша паразиты	4	Humpback salmon parasites
Горбуша инфекции	0	Humpback salmon infections
Карповые паразиты	24	Carp parasites
Карповые инфекции	4	Carp infections
**FISH TOTAL matches**	**820**	
Selected for initial full-text analysis	48<br>5	in Fish Parasites, 1997-2018<br>in Fish Infections, 2010 – 2018
Included in the Article Table (excel) after brief abstract-text analysis	47<br>2	in arctic regions<br>in Russia total/random regions (might be still relevant)<br>1997 - 2018

MAMMALS:

Search term in Russian	Matches	Search term translated to English
Инфекционные болезни животных	505	Infectious diseases animals
Инфекции животных<br>plus Арктика<br>or plus	1190<br>3<br>36	Infections animals<br>plus Arctic<br>plus northern
Инфекционные болезни млекопитающих	19	Infectious diseases mammals
Инфекции млекопитающих	105	Infections mammals
Инвазивные болезни животных	0	Invasive diseases animals
Инвазии животных	485	Invasions animals
Инвазивные болезни млекопитающих	0	Invasive diseases mammals

Search Terms Used:

Инвазии млекопитающих	38	Invasions mammals
Болезни морских млекопитающих	7	Diseases marine mammals
**MAMMALS TOTAL matches**	**2388**	
Selected for initial full-text analysis	88	1997-2018
Included in the Article Table (excel) after brief abstract-text analysis	71<br>14	in arctic regions<br>in Russia total/random regions (might be still relevant)<br>2007 - 2018

Some additional search information by infection

Key words	Mammals	Animals	Bear	Reindeer	Fox	Hare	Wolf	Sled dog	*Matches*	*Selected for full-text analysis*	*Items selected, years range*
Trichinella<br>Трихинелла	19	43	10	-	3	-	10	11	115	*24*	*2007-2017*
Toxoplasma<br>Токсоплазма	-	1	-	-	-	-	-	5	14	*1, no full-text*	*1997*
Anthrax<br>Сибирская язва	1	148	-	22	-	-	-	-	422	*29*	*2011-2018*
Tularemia<br>Туляремия	33	42	-	-	-	3	-	4	328	*11*	*2015-2018*
Other infections*										*23*	*2008-2018*

Other generic searches

Search term in Russian	matches	Search term translated to English
Инфекции в живой природе	7	Infections in wildlife
Инфекции в дикой природе	12	Infections in wildlife
Инфекции в животном мире	52	Infections in animal world
Инфекции в фауне	30	Infections in fauna

Included in the Article Table (Other_Rus page in excel) after abstract-text analysis: 3, 2009 – 2017
