# Supplementary material for: Research on Selected Wildlife Infections in the Circumpolar Arctic—A Bibliometric Review
Source: Int J Environ Res Public Health. 2022 Sep 7;19(18):11260. doi: 10.3390/ijerph191811260 (PMC9517571; doi:10.3390/ijerph191811260)
Supplement: Supplementary file 1 [file ijerph-19-11260-s001.zip › Table S1.pdf]

Table S1. Number of articles published by English language journals (n = 77). The total number of included articles is 228.

Journal	Number of articles
Journal of Wildlife Diseases	48
Veterinary Parasitology	17
Parasitology Research	13
Journal of Parasitology	10
PLoS ONE	9
Parasitology	8
Veterinary Record	7
Parasites & Vectors	6
Acta Veterinaria Scandinavica	4
Archives of Virology	4
Canadian Journal of Zoology	4
Diseases of Aquatic Organisms	4
Journal of Veterinary Diagnostic Investigation	4
Virology Journal	4
Avian Diseases	3
Emerging Infectious Diseases	3
IJP: Parasites and Wildlife	3
Journal of Fish Diseases	3
Oikos	3
Polar Biology	3
European Communicable Disease Bulletin	2
Hydrobiologia	2
Infection, Genetics and Evolution	2
Journal of Clinical Microbiology	2
Journal of Icthyology	2
Journal of the American Veterinary Medical Association	2
Polar Research	2
Research in Veterinary Science	2
Veterinary Microbiology	2
Veterinary Research	2

Veterinary Research Communications	2
Acta Parasitologica	1
American Journal of Tropical Medicine and Hygiene	1
Aquaculture	1
Berliner und Munchener tierärztliche Wochenschrift	1
BMC Veterinary Research	1
Bulletin- European Association of Fish Pathologists	1
Canadian Field Naturalist	1
Canadian Journal of Fisheries and Aquatic Sciences	1
Canadian Journal of Microbiology	1
Canadian Veterinary Journal	1
Comparative Immunology, Microbiology and Infectious Diseases	1
Comparative Parasitology	1
Cross-Canada Disease Report	1
EcoHealth	1
Ecological Applications	1
Epidemiology and Infection	1
Food Anal. Methods	1
Foodborne Pathogens and Disease	1
Functional Ecology	1
Helminthologia	1
Integrative Zoology	1
International Journal for Parasitology	1
Journal of Animal Ecology	1
Journal of Applied Microbiology	1
Journal of Fish Biology	1
Journal of Food Protection	1
Journal of General Virology	1
Journal of Helminthology	1
Journal of Medical Entomology	1
Journal of Veterinary Medicine	1
Kleintierpraxis	1
Marine Biology Research	1
Med Parazitol (Mosk).	1
Mem Inst Oswaldo Cruz	1
Parazitologiia	1

Polar Science	1
Polish Polar Research	1
Rev. Sci. Tech.	1
The Royal Society	1
Transboundary and Emerging Diseases	1
Ugeskrift for laeger	1
Vector-Borne and Zoonitic Diseases	1
Veterinary Dermatology	1
Virology	1
Zoologicheskii Zhurnal	1
Zoonoses Public Health	1
Total number of articles	228
