# Supplementary material for: Research on Selected Wildlife Infections in the Circumpolar Arctic—A Bibliometric Review
Source: Int J Environ Res Public Health. 2022 Sep 7;19(18):11260. doi: 10.3390/ijerph191811260 (PMC9517571; doi:10.3390/ijerph191811260)
Supplement: Supplementary file 1 [file ijerph-19-11260-s001.zip › Table S2.pdf]

Table S2. Number of articles published by Russian language journals (n = 60). The total number of included articles is 122.

Journals	Number of articles
Паразитология	17
Проблемы Особо Опасных Инфекций	7
Российский Паразитологический Журнал	7
Достижения Науки И Техники Апк	5
Научный Вестник Ямало-Ненецкого Автономного Округа	5
Сибирский Вестник Сельскохозяйственной Науки	5
Актуальные Вопросы Ветеринарной Биологии	3
Зоологический Журнал	3
Исследования Водных Биологических Ресурсов Камчатки И Северо-Западной Части Тихого Океана	3
Международный Вестник Ветеринарии	3
Аграрная Наука Евро-Северо-Востока	2
Вестник Бурятской Государственной Сельскохозяйственной Академии Им. В.р. Филиппова	2
Вестник Северо-Восточного Научного Центра Дво Ран	2
Вестник Сургутского Государственного Университета	2
Вестник Ульяновской Государственной Сельскохозяйственной Академии	2
Генетика И Разведение Животных	2
Дальневосточный Журнал Инфекционной Патологии	2
Известия Санкт-Петербургского Государственного Аграрного Университета	2
Международный Научно-Исследовательский Журнал	2
Наука И Образование: Современные Тренды	2
Российский Журнал Биологических Инвазий	2
Теория И Практика Борьбы С Паразитарными Болезнями	2
Труды Карельского Научного Центра Российской Академии Наук	2
Ученые Записки Петрозаводского Государственного Университета	2
Acta Biomedica Scientifica	1
Аграрный Вестник Урала	1
Биология Моря	1
Бюллетень Физиологии И Патологии Дыхания	1
Вестник Апк Ставрополья	1

Вестник Государственного Аграрного Университета Северного Зауралья	1
Вестник Казанского Государственного Энергетического Университета	1
Вестник Камчатского Государственного Технического Университета	1
Вестник Красноярского Государственного Аграрного Университета	1
Вестник Мурманского Государственного Технического Университета	1
Вестник Новосибирского Государственного Университета. Серия: Биология, Клиническая Медицина	1
Вестник Тюменского Государственного Университета. Экология И Природопользование	1
Вопросы Нормативно-Правового Регулирования В Ветеринарии	1
Евразийское Научное Объединение	1
Журнал Инфектологии	1
Известия Российской Академии Наук. Серия Биологическая	1
Известия Самарского Научного Центра Российской Академии Наук	1
Медицина Кыргызстана	1
Наука И Мир	1
Наука И Образование	1
Научные Труды Дальневосточного Государственного Технического Рыбохозяйственного Университета	1
Пермский Аграрный Вестник	1
Проблемы Экологического Мониторинга И Моделирования Экосистем	1
Профилактическая И Клиническая Медицина	1
Российский Ветеринарный Журнал. Мелкие Домашние И Дикие Животные	1
Российский Журнал Проблемы Ветеринарной Санитарии, Гигиены И Экологии	1
Современные Проблемы Науки И Образования	1
Современные Тенденции Развития Науки И Технологий	1
Технологии Гражданской Безопасности	1
Труды Вниро	1
Успехи Современной Биологии	1
Ученые Записки Казанской Государственной Академии Ветеринарной Медицины Им. Н.э. Баумана	1
Фундаментальные И Прикладные Исследования В Современном Мире	1
Хранение И Переработка Сельхозсырья	1
Экология	1
Якутский Медицинский Журнал	1
Total number of included articles	122
