# Supplementary figures and images for: Research on Selected Wildlife Infections in the Circumpolar Arctic—A Bibliometric Review
Source: Int J Environ Res Public Health. 2022 Sep 7;19(18):11260. doi: 10.3390/ijerph191811260 (PMC9517571; doi:10.3390/ijerph191811260)

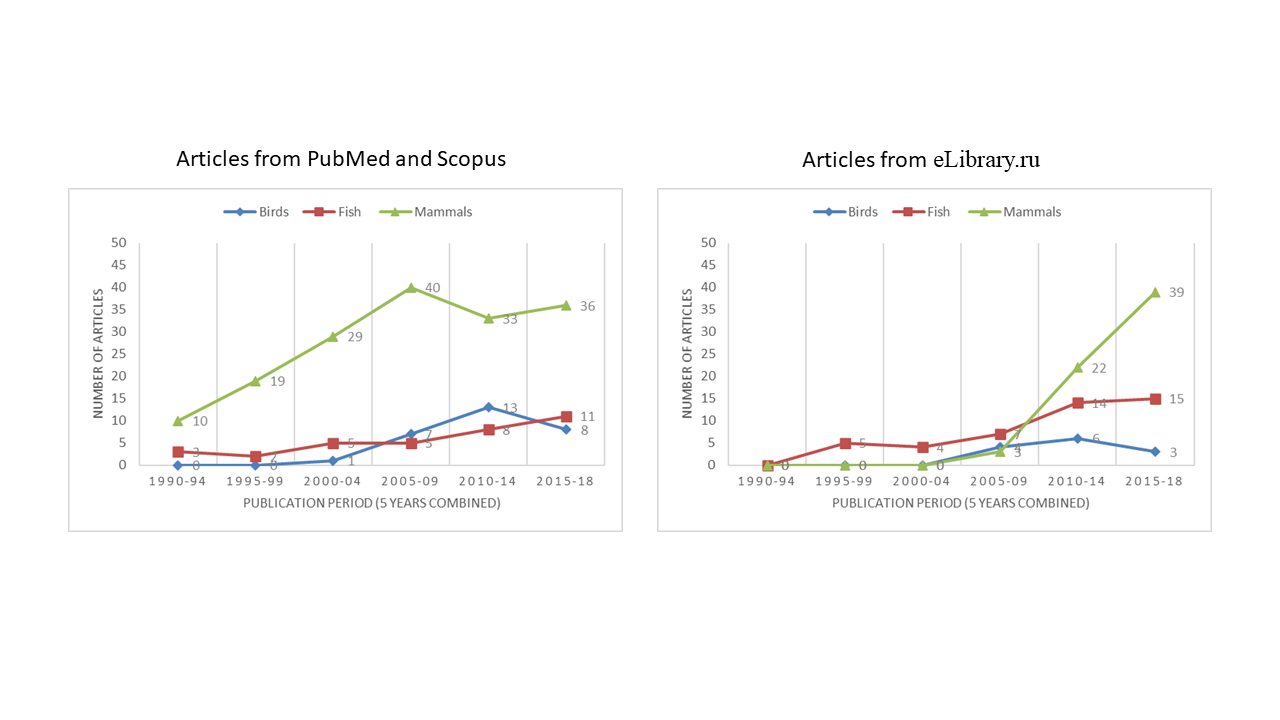

Supplement: Supplementary file 1 [file ijerph-19-11260-s001.zip › Fig S1.tif]
